# Turning the Table: Plants Consume Microbes as a Source of Nutrients

**DOI:** 10.1371/journal.pone.0011915

**Published:** 2010-07-30

**Authors:** Chanyarat Paungfoo-Lonhienne, Doris Rentsch, Silke Robatzek, Richard I. Webb, Evgeny Sagulenko, Torgny Näsholm, Susanne Schmidt, Thierry G. A. Lonhienne

**Affiliations:** 1 School of Biological Sciences, The University of Queensland, Brisbane, Queensland, Australia; 2 Institute of Plant Sciences, University of Bern, Bern, Switzerland; 3 The Sainsbury Laboratory, Norwich Research Park, Norwich, United Kingdom; 4 Centre for Microscopy and Microanalysis, The University of Queensland, Brisbane, Queensland, Australia; 5 School of Biochemistry and Molecular Biosciences, The University of Queensland, Brisbane, Queensland, Australia; 6 Department of Forest Ecology and Management, Swedish University of Agricultural Sciences, Umeå, Sweden; CNRS UMR 8079/Université Paris-Sud, France

## Abstract

Interactions between plants and microbes in soil, the final frontier of ecology, determine the availability of nutrients to plants and thereby primary production of terrestrial ecosystems. Nutrient cycling in soils is considered a battle between autotrophs and heterotrophs in which the latter usually outcompete the former, although recent studies have questioned the unconditional reign of microbes on nutrient cycles and the plants' dependence on microbes for breakdown of organic matter. Here we present evidence indicative of a more active role of plants in nutrient cycling than currently considered. Using fluorescent-labeled non-pathogenic and non-symbiotic strains of a bacterium and a fungus (*Escherichia coli* and *Saccharomyces cerevisiae*, respectively), we demonstrate that microbes enter root cells and are subsequently digested to release nitrogen that is used in shoots. Extensive modifications of root cell walls, as substantiated by cell wall outgrowth and induction of genes encoding cell wall synthesizing, loosening and degrading enzymes, may facilitate the uptake of microbes into root cells. Our study provides further evidence that the autotrophy of plants has a heterotrophic constituent which could explain the presence of root-inhabiting microbes of unknown ecological function. Our discovery has implications for soil ecology and applications including future sustainable agriculture with efficient nutrient cycles.

## Introduction

Plants and microbes have evolved detrimental and beneficial relationships. Detrimental relationships involve pathogens including fungi, bacteria and viruses [1] and the hallmark of pathogenic interactions is the suppression and interference with plant immune responses [2], [3]. Beneficial relationships include symbiosis [1], diazotrophic endophytes that supply the plant with fixed nitrogen [4], [5] and other endophytic associations that promote plant growth by producing phytohormones, volatiles, defence compounds, and enzymes [6], [7], [8], [9], [10]. A less well-defined beneficial relationship involves the association of plant roots with microbes in the rhizosphere. Roots attract soil microbes by exuding nutrient sources including carbohydrates, organic and amino acids [11], [12], [13], [14] and the density of microbes in the rhizosphere is much higher than in bulk soil [15]. According to the “soil microbial loop” concept, nutrients and carbon are cycled between soil and microbial pools [16], [17], [18], and inorganic and organic nutrients of low molecular mass become available through microbial turnover of soil organic matter and are subsequently ‘scavenged’ by the plant root.

However, new concepts are emerging which point to a wider range of nutrient sources for plants [19] and question the ‘soil microbial loop’ concept. We recently demonstrated that roots can incorporate large organic molecules including proteins and DNA [20], [21], and this implies that plants may be less dependent on microbial activity for break-down of organic matter than currently assumed. Adding to mounting questions of plant-microbe interactions in soil is the discovery that diverse microbes without known relationships with plants exist in roots [22].

Here, we explored the possibility that plants take up and digest microbes as a source of nutrients. We discovered that Arabidopsis (*Arabidopsis thaliana*) and tomato (*Lycopersicum esculentum*) are able to take up non-pathogenic *E. coli* and *S. cerevisiae* into root cells, digest and use these microbes as a nutrient source. Our results show that the uptake process involves modification of the walls of root cells which is followed by active incorporation and degradation of the incorporated microbes.

## Results and Discussion

### Bacteria and yeast are taken up by Arabidopsis and tomato

**Figure 1:**
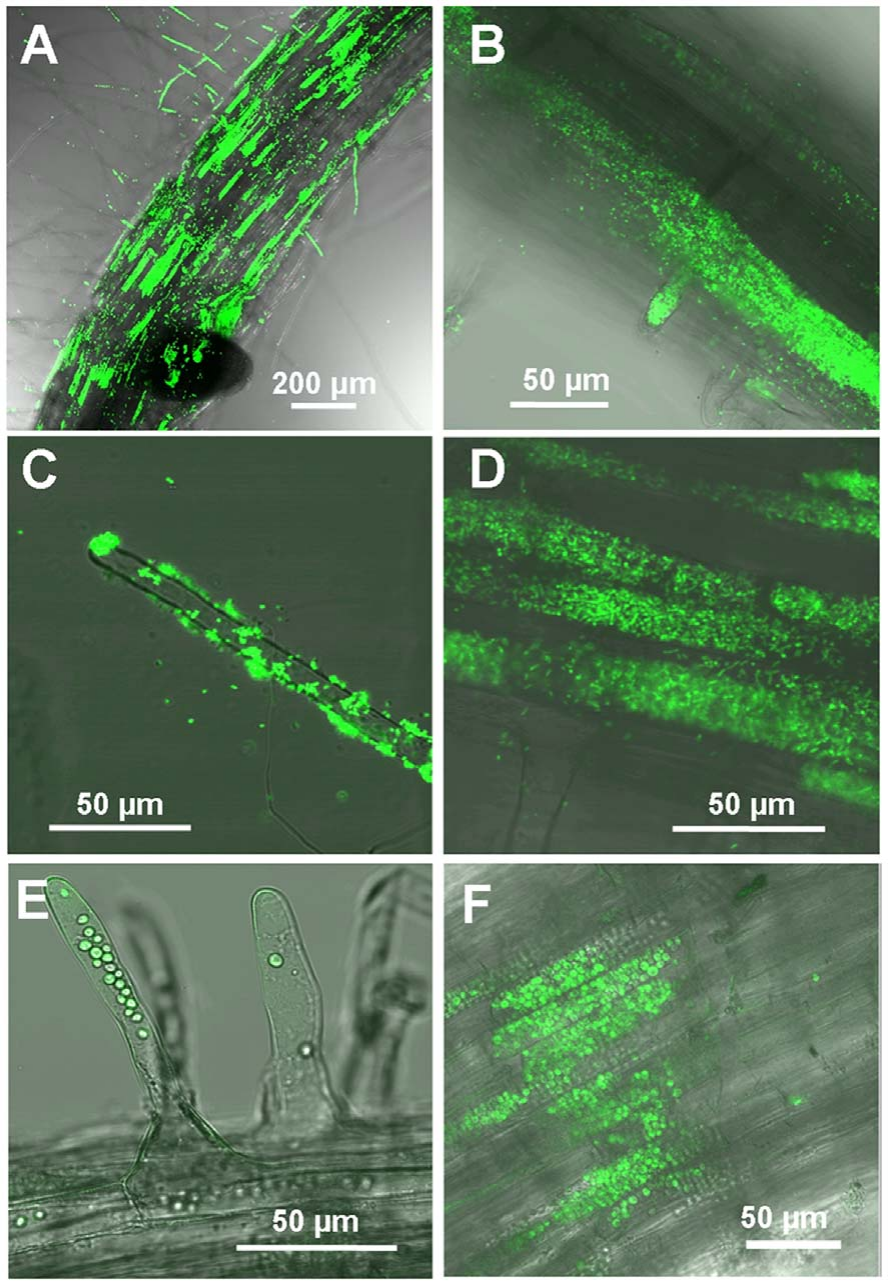
Roots of axenically grown Arabidopsis and tomato were incubated with *E coli* or yeast expressing green fluorescent protein (^GFP^ *E. coli* or ^GFP^yeast). ^GFP^ *E. coli* was detected at the surface of roots and root hairs (A and C), and inside roots and root hairs (B and D). ^GFP^Yeast was present inside roots and root hairs (E and F). (A, D and F) and (B, C and E) correspond to tomato and Arabidopsis root, respectively. Fluorescent images were taken by confocal laser scanning microscopy (CLSM).

To examine if plants take up microbes and use them as a nutrient source, we incubated roots of intact Arabidopsis and tomato plants with *E. coli* Bl21 and yeast *S. cerevisiae* which express the green fluorescent protein (^GFP^ *E. coli* and ^GFP^yeast). To examine plants with different root specialisations, we chose Arabidopsis which does not form symbiotic relationships and tomato which forms symbioses with mycorrhizal fungi, but was grown here without symbionts. Plants were cultivated in non-axenic hydroponic (tomato) and axenic agar (Arabidopsis) culture. Microbial solution was added to growth media ensuring that roots were not disturbed or damaged. After 12 h (tomato) or 4 h (Arabidopsis) incubation, ^GFP^ *E. coli* and ^GFP^yeast were detected in root hairs and the rhizodermis and cortex of mature zones of the roots (^GFP^ *E. coli*, Figure 1A–D; yeast, Figure 1E–F) by confocal laser scanning microscopy (CLSM). Cytoplasmic streaming in root hairs (^GFP^ *E. coli*: movie S1; ^GFP^yeast: movie S2) and other root cells (movie S3) was indicative of live and active plant cells. Similar results were obtained with Arabidopsis axenic hydroponic culture and soil-grown Arabidopsis or tomato. To demonstrate the specificity of the uptake process, we incubated Arabidopsis with 5 µm nano-silica fluorescent beads similar in size to yeast (3–5 µm) but larger than *E. coli* (<2 µm). No beads were detected in roots and few beads were attached to root surface after washing (Figure S1) suggesting that roots recognize microbes and this results in targeted incorporation.

**Figure 2:**
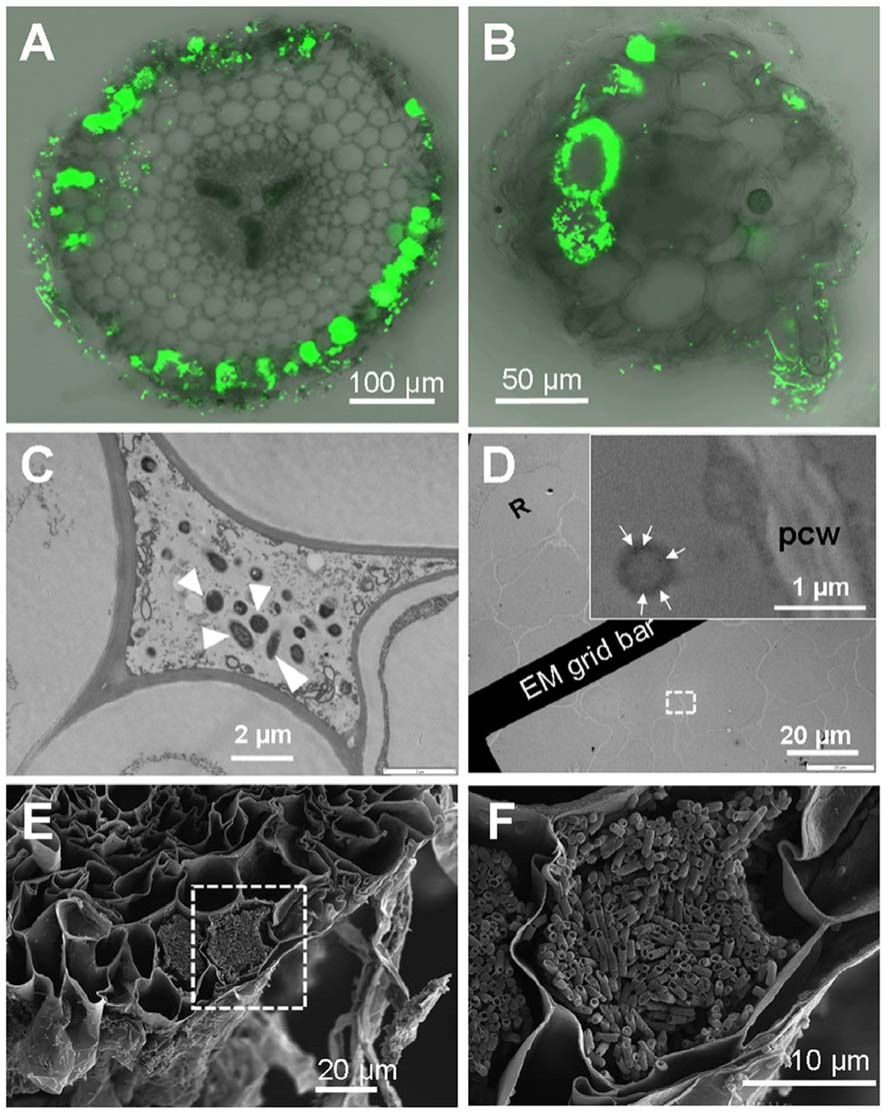
Root transverse sections and electron micrographs of tomato and Arabidopsis show ^GFP^ *E. coli* in the apoplast and inside root cells. *E. coli* was detected inside tomato roots (A, C and D, E and F) and Arabidopsis roots (B). (A and B) Fluorescent images of transverse sectioned roots taken by CLSM. (C and D) Images taken by a transmission electron microscope. White triangles in (C) indicate *E. coli* cell present in apoplast. (D) Roots were probed with immunogold-labeled anti-GFP revealing *E. coli* in root cortex cells. Sub-image in (D) is a detail of dash-white square box. Gold labeling is marked with white arrows. Rhizodermis cell (R) and plant cell wall (pcw) is indicated. (F) is a detail image of (E) showing plant cells containing *E. coli*, and both images were taken by SEM.

CLSM of root cross-sections revealed microbes were present in epidermis cells, cortex cells and the apoplastic space, but absent from tissue separated by the Casparian strip (Figure 2A and B; Figure S2; movie S4 and S5). Transfer of bacteria from root surfaces was avoided by coating roots with agar prior to processing. Transmission Electron Microscopy (TEM) verified that cells of *E. coli* Bl21 were present in the intercellular space (Figure 2C) and inside cortex cells (Figure 2D). This finding was confirmed with Scanning Electron Microscopy (SEM) showing *E. coli* Bl21 in epidermis cells (Figure 2E and F) and demonstrates that non-pathogenic and non-symbiotic microbes enter cells of mature roots.

### After uptake, microbes are confined to root cortex cells where they are degraded

**Figure 3:**
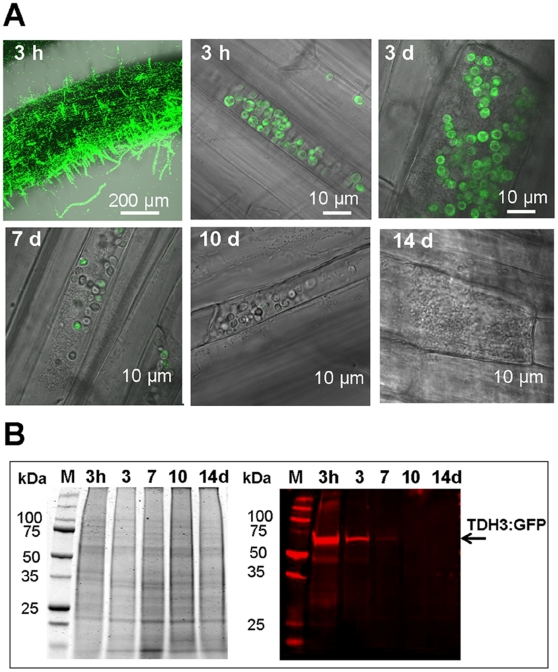
Time course experiment of yeast degradation in tomato roots. (A) The number of living yeast cells (fluorescing green) in tomato decreased over time as observed by CLSM. (B) The amount of recombinant TDH3:GFP protein present inside the roots 0decreased over time. Equal amounts of proteins from root extracts were separated by SDS-PAGE (B, Left) and analyzed by western blot using anti-GFP antibody to detect yeast recombinant TDH3:GFP protein (B, Right).

We investigated the fate of microbes after incorporation into root cells. Hydroponic tomato plants were incubated overnight with ^GFP^yeast. Since expression of GFP in yeast clone *TDH3* (YGR192C) is constitutive, monitoring of GFP fluorescence allows an assessment of yeast cells activity. Three hours after incubation, fluorescing ^GFP^yeast cells were detected at the root surface and inside root cells (Figure 3A). After 3 days, ^GFP^yeast was only detected inside roots, with some yeast cells alive and fluorescing, and some non-fluorescing yeast cells displaying an altered shape (Figure 3A). Few yeast cells were fluorescing after 7 days, no GFP signal was detected after 10 days, and root cells contained only debris of yeast cells after 14 days (Figure 3A). To support microscopy findings, we quantified the TDH3:GFP fusion protein (expressed constitutively by ^GFP^yeast) in roots harvested in parallel with CLSM-inspected plants by western blot analysis (Figure 3B). TDH3:GFP in roots strongly diminished over time (Figure 3B). No protein was detected in roots after 10 and 14 days incubation, confirming CLSM findings.

Over the time of the experiment, tomato plants retained a healthy phenotype. To confirm that *E. coli* Bl21 was not a threat to plants, we incubated Arabidopsis grown on MS medium with *E. coli* Bl21 and monitored growth for 14 days. Similarly to tomato, Arabidopsis plants grown with or without *E. coli* Bl21 had similar appearance (Figure S3).

CLSM analysis revealed no evidence that ^GFP^ *E. coli* or ^GFP^yeast were transported to leaves. The absence of microbes in leaves was confirmed as leaf homogenates incubated on LB media containing selective antibiotics did not produce colonies. Similarly, incubation of Arabidopsis roots with *Salmonella typhimurium* caused proliferation of *Salmonella* in root cells, but not leaves [23].

Taken together, our results demonstrated that upon incorporation, *E. coli* Bl21 and yeast are confined to the root cells were they are degraded.

### *E. coli* Bl21 induces cellulase(s) activity in roots of *Arabidopsis*

A central question is how *E. coli* and yeast enter intact root cells. Plant cells possess walls composed of a highly integrated and structurally complex network that acts as barrier to larger molecules, particles and microbes [24]. We assumed that *E. coli* Bl21 and yeast can only enter intact root cells if cell walls are degraded prior to entry. Pathogens attack cell walls by secretion of polysaccharide-degrading enzymes including polygalacturonases and cellulases [25], and *Rhizobium* infection in the legume root symbiosis occurs through the activity of a cell-bound bacterial cellulase [26]. There are no reports of non-pathogenic and non-symbiotic microbes degrading plant cell walls.

**Figure 4:**
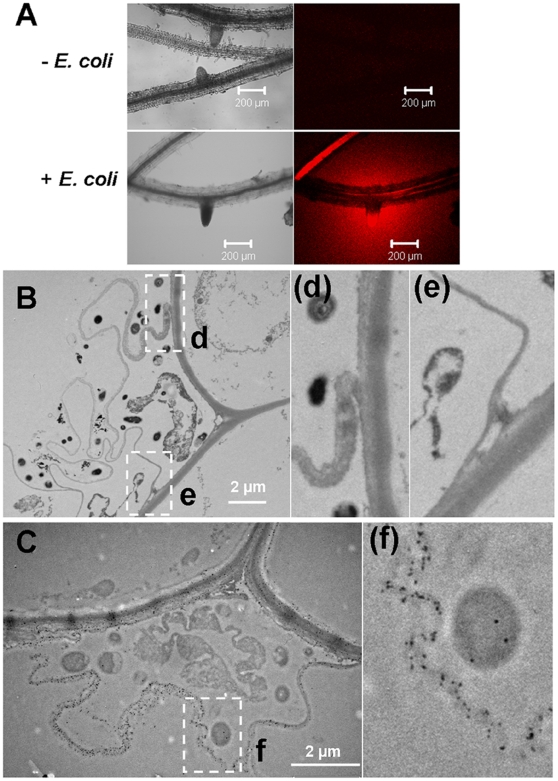
Root produced cellulase and extended the cell wall when incubated with *E. coli* Bl21. (A) Incubation of Arabidopsis roots in 31 µg/mL resorufin cellubioside after incubating overnight with *E. coli*. After 2 h incubation, roots were viewed by CLSM. (B) TEM image of cell wall-like structure of plant roots encompassing bacteria. (C) TEM image of cellulase-gold labeling on the root sections with double labeling with the anti-GFP antibody. The size of the gold particle on bacteria is 15 nm (Au-particle specific to ^GFP^ *E. coli*) and gold particles on the plant material are 10 nm (Au-particle specific to plant cellulose). (d), (e) and (f) are detail images of insets d, e and f.

We therefore examined whether plant-derived cell wall degrading enzymes facilitate entry of the microbes studied here. Hydroponic Arabidopsis were incubated overnight with *E. coli* Bl21 and transferred to liquid MS medium containing resorufin-β-D-cellobioside (Res-CB), an artificial substrate for cellulases that emits red fluorescence upon cleavage[27]. Fluorescence increased in *E. coli* Bl21-incubated roots (Figure 4A) but not in *E. coli* incubated with Res-CB or roots grown without *E. coli*. The assay does not allow localization of the origin of cellulase activity because the generated resorufin diffuses rapidly through tissues. These results indicate that the presence of *E. coli* in the medium triggers the induction of cellulase expression in Arabidopsis, and this may be linked to the uptake of *E. coli* into root cells.

### *E. coli* Bl21 induces plant cell wall-like outgrowth

Additional insights into the mechanisms of microbial uptake were obtained by TEM. Clusters of *E. coli* Bl21 at the surface of root cells were systematically surrounded by a thin layer of an undetermined structure. This layer resembled the structure that was equivocally reported to be a matrix-like bacterial substance suggestive of a site from which bacterial cells may gain entry into young roots [28] or mucilaginous material secreted either by plants or bacteria for binding bacteria to the root surface [29]. Our results suggest that this structure consists of cell wall components as it was connected to the cell wall of the rhizodermis (Figure 4B). Dual gold (Au)-labeling of sections with Au-labeled cellulase (10 nm) and Au-labeled anti-GFP antibody (15 nm) showed that this structure is at least partly composed of cellulose (Figure 4C), and indicate that Arabidopsis synthesizes a cell wall-like structure that contains cellulose. Thus, a step in the process of the acquisition of microbes by roots may involve ‘corralling’ microbes at the root surface by cell wall-like outgrowth for subsequent incorporation. This sophisticated mechanism has interesting connotations with the mechanism used by *Agrobacterium tumefaciens* to adhere to the root surface of plants for infection. During the infection process, *Agrobacterium* produce cellulose fibrils via the activity of its own cellulose synthases to strengthen its adherence to the surface of the roots [30].

### *E. coli* Bl21 triggers extensive alteration of the expression of genes involved in cell wall modification

**Figure 5:**
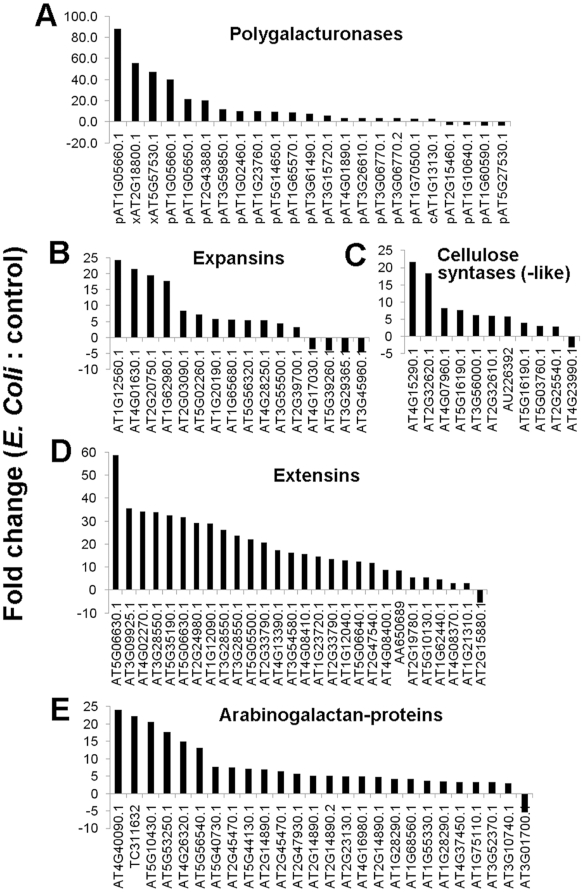
Arabidopsis genes involved in cell wall modification with differential expression at the time incubated with *E coli* Bl21 compared with control. Gene expression more than 3 fold changes were shown. (A) Glycosyl hydrolases and lyases (x, c, p are the symbol for xyloglucan endotransglycosylase, cellulase and pectinases/putative pectinases, respectively). (B) Expansins. (C) Cellulose syntases and cellulose syntase-like. (D) Extensins. (E) Arabinogalactan proteins.

To further explore mechanisms involved in the observed plant-microbe interactions, we proceeded with genome-wide transcriptome analysis of Arabidopsis roots incubated with *E. coli* Bl21 for 24 hours. Microarray data revealed that a numerous number of genes involved in cell wall modification increased in expression (Figure 5). Strongly induced were the expression of cellulases (endo-glucanases) and other cell wall degrading enzymes including pectinases and xyloglucan endotransglycosidases (Figure 5A), supporting our biochemical analysis (Figure 4A). Expression of expansins, involved in cell wall loosening, was also highly up-regulated (Figure 5B). Consistent with EM data demonstrating cell wall-like outgrowth containing cellulose (Figure 4B and C), cellulose synthases, cellulose synthases-like, and extensins were strongly up-regulated (Figure 5C*–*D). Cellulose synthase-like proteins (CLSs) are involved in the linkage of non-cellulosic polysaccharides [31]. The induction of cellulose synthase-like genes (*CSLs*) observed here is interesting in the view that mutation of *CSLA9* (At5G03760) in Arabidopsis leads to inhibition of *Agrobacterium*-mediated root transformation through reduced ability of the roots to bind *A. tumefaciens* [32]. Zhu et al. (2003) did not observe any major differences in the linkage structure of the non-cellulosic polysaccharides in the *CLSA9* defective mutant and hypothesized that the defect in binding A. *tumefaciens* may arise from the altered ability of the Arabidopsis mutant to secrete particular polysaccharides necessary for bacterial recognition of the host and subsequent attachment. Our results suggest that CLSs may be involved in the recognition and attachment of *E. coli* Bl21 at the root surface. Extensins are also potential candidates for such function because they have been reported to be involved in similar processes. Cannon et al. (2008) [33] have established that extensins are involved in the formation of the cross wall (cell plate) during cytokinesis and they proposed that self-assembling extensins serve as scaffolds for ordered pectin deposition in the cell plate. It is reasonable to conceive that the extensins that are over expressed in reaction of *E. coli* Bl21 treatment have a role in the cell wall-like outgrowth.

In relation to microbe attachment at the surface of the roots, the over-expression of arabinogalactan-proteins (Figure 5E) involved in formation of wall ingrowths [34] is also likely to be relevant. Mutation of arabinogalactan-protein AGP17 (At2G23130) in Arabidopsis resulted in decreased efficiency of *Agrobacterium*-induced transformation due to altered binding to the root surface caused by reduced direct binding or impaired signaling pathway(s) [35]. The strong induction of nearly all arabinogalactan-genes of Arabidopsis treated with *E. coli* Bl21 (Figure 5E) corroborates with arabinogalactan proteins promoting binding of microbes to the root surface.

Further, it is possible that endocytosis or a related process is involved in the incorporation of microbes into root cells. Induction of genes involved in cytoskeleton structure and re-organization (Figure S4) supports this hypothesis.

Altogether, our results indicate that uptake of microbes by roots occurs through major structural modifications of root cells controlled by the plant, including outgrowth of a cell-wall like structure capturing microbes, and degradation and/or loosening of cell walls with plant-derived enzymes. In contrast, entry of pathogenic and symbiotic microbes into root cells is controlled predominantly or partly by microbes; pathogenic fungi enter plants by secreting enzymes that degrade plant cell walls [36], and infection of legume root cells with symbiotic rhizobia requires formation of special root hairs initiated by rhizobia-secreted Nod factors [37]. The process of colonization of roots by diazotrophic endophytes is also fundamentally different from our observations in mature roots, because diazotrophic endophytes enter in elongation zones and through cracks at the point of lateral roots emergence [4], [38], [39]. Although root colonization by diazotrophic endophytes involves cell wall degradation processes, the source of the cell-wall degrading enzymes differs. Diazotrophs release plant cell-wall-degrading enzymes for the ingress into roots [40], [41], whereas plant-derived cell-wall degrading enzymes facilitated entry of *E. coli* and yeast into mature roots. Thus, the uptake of *E. coli* and yeast involves mechanisms which have not been described by previous research, further indicating that the observed processes are hitherto un-described interactions between microbes and plants.

### *E. coli* Bl21 is a nitrogen source for plants

**Figure 6:**
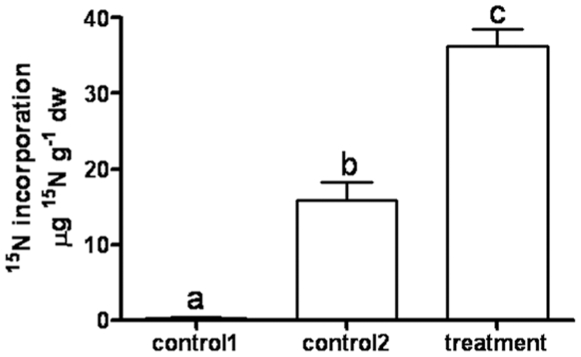
Incorporation of *E. coli*-derived ^15^N by leaves of tomato plants. Roots of tomato grown in hydroponic culture were incubated with ^15^N-*E. coli* for 1 h. After washing of the roots, plants were further grown for 2 weeks. Then 2–3 new leaves were analyzed for ^15^N content. Control 1 are plants grown without ^15^N-*E. coli*. Control 2 are plants incubated for 2 h with filtered ^15^N-*E. coli* incubation solution. Results are depicted as mean ± SD (n = 7). Different letters indicate significant differences at *p<0.001* (1-way ANOVA, Tukey's posthoc test).

To determine whether microbes are a nutrient source for plants, we incubated roots of hydroponic tomato plants for 1 h with ^15^N-labelled *E. coli* Bl21 (^15^N-*E. coli*) and analyzed new leaves for ^15^N content. Controls included plants not incubated with *E. coli* and plants incubated with filtrate of ^15^N-*E. coli* solution to account for possible ^15^N release from bacteria during incubation. Plants were rinsed and grown hydroponically for 2 weeks. New leaves of ^15^N-*E.coli*-incubated plants had a significantly higher concentration of ^15^N than controls (Figure 6). Although this experiment does not provide unequivocal evidence that *E. coli* is digested inside root cells, it demonstrates that nitrogen derived from *E. coli* is assimilated by plants.

### General considerations

Our experiments show that in the absence of pathogenic or symbiotic relationships, plants coordinated the entry of *E. coli* and yeast into root cells with an apparent expenditure of energy that is most likely justified by the benefit of using microbes as a nutrient source.

It appears that the pronounced plant responses to exposure to microbes, including induction of gene expression and remodeling of cell walls, is highly localized and strictly regulated to minimize the cost for the plant. It is possible that plant responses to non-pathogenic microbes are controlled at the cellular level, and evidence for this suggestion is provided by the patchiness of microbial uptake in the mature root zones (Figure 1A). We show that the presence of microbes induces the expression of plant enzymes with divergent functions, such as cellulases and cellulose synthases, and this suggests that the uptake process consist of a succession of distinct and tightly regulated processes, which would exclude the possibility of permanent induction of genes. In addition to minimizing energy expenditure, a transient and localized uptake process would also reduce opportunities for pathogens to invade the root.

Adding to the energetic costs of the uptake process, possible loss of turgor and cell contents could be associated with the entry of microbes into root cells. It is conceivable that one of the functions of the observed cell wall outgrowth limits loss of cell contents by preventing diffusion and leakage into the rhizosphere. Future research has to scrutinize the observed processes including each step of incorporation and digestion, and how nutrients gains relate to energy expenditures and possibly loss of cell content.

Our discovery may explain the high diversity of root-inhabiting microbes of unknown ecological function [22], [42] and brings a new dimension to current concepts of rhizosphere ecology. Much attention has focused on plant-growth-promoting bacteria for their potential to enhance plant growth [10], [43]. Our discovery indicates the presence of a further category of plant-growth-promoting microbes which are used as a direct nutrient source. It is tempting to speculate that the microbe-enriched rhizosphere maintained by plants through exudation of photosynthates [16] is in part a ‘microbe nursery’ facilitating direct nutrients supply to plants.

Mixotrophy, the use of nutrients derived from photosynthesis and organic sources, is considered an exception in higher plants but characteristic of photosynthetic phytoplankton [44]. Our results indicate that mixotrophy may also occur in higher plants. This discovery has implications for carbon, nitrogen and phosphorus cycles in soils. High-production crop systems carry a strong pollution footprint which contributes to greenhouse gas emissions and pollutes ground and surface waters [45], and new approaches to supply soil-derived nutrients efficiently to plants are being sought. Exploiting the synergistic interactions between plants and microbes by harnessing soil microbes to supply crops with nutrients may be a further strategy.

## Materials and Methods

### Plasmid Construction

The *green fluorescent protein (GFP)* coding region was cloned as a glutathione S-transferase (GST) tagged recombinant gene. GFP was amplified from pDH51-GW-EGFP (GenBank: AM773753.1) using the following forward and reverse primers: 5′- GGC TCG AGA TGG TGA GCA AGG GCG AGG AG-3′ and 5′- GGA AGC TTT CAC TTG TAC AGC TCG TCC ATG CC -3′. The PCR product was digested with XhoI and HindIII and cloned into pGEX-KG [46] designated pGSTGFP.

### Preparation of *E. coli* Expressing GFP

*E. coli* strain Bl21 (DE3) (Novagen) carrying the plasmid pGTf2 (TAKARA BIO INC) was transformed with pGSTGFP. Recombinant *E. coli* cells were selected on LB plates containing 200 µg ml^−1^ ampicillin and 35 µg ml^−1^ chloramphenicol. A single colony was used to inoculate a pre-culture containing 20 ml of LB supplemented with ampicillin (200 µg ml^−1^) and chloramphenicol (35 µg ml^−1^). The pre-culture was grown overnight at 37°C and used to inoculate 1 l of LB supplemented with ampicilin and chloramphenicol. *E. coli* was grown at 37°C to a cell density of 0.6 to 1 *A*_600_ units. Cells were cooled down on ice and 1 mM of Isopropyl-1-thio-β-D-galactopyranoside (IPTG) was added to induce expression of GFP. After incubation on a shaker (160 rpm) for 16–20 hours at 18°C, cells were harvested by centrifugation and washed twice with 1 l of 5 mM MES pH 5.8 (wash buffer) and resuspended in wash buffer. Cultures were used immediately.

### Preparation of *Saccharomyces cerevisiae* Expressing GFP

^GFP^yeast clone *TDH3* (YGR192C) (Invitrogen, California, USA) expressing glyceraldehyde-3-phosphate dehydrogenase fused to GFP was selected among the entire ^GFP^yeast clone library for its high emission of green fluorescence (yeastgfp.yeastgenome.org). A single colony was used to inoculate 1 l of Yeast-extract Peptone Dextrose (YPD) liquid media and the culture was grown for 48 h at 28°C. Cells were harvested by centrifugation, washed twice with 1 l of wash buffer and re-suspended in wash buffer. Cultures were used immediately.

### Plant Growth Conditions

Arabidopsis (*Arabidopsis thaliana* ecotype *Columbia* [Col-0]) plate culture: Seeds were germinated axenically on Petri dishes containing Murashige and Skoog (MS[47]) medium solidified by 3.2 g l^−1^ of phytagel (Sigma). Plates were positioned vertically so that germinating radicals grow downward along the gel surface. Plants were grown for 2–3 weeks in a growth room with 16/8 h light/dark, 21°C, 150 µmol m^−2^ s^−1^ light intensity Arabidopsis axenic hydroponic culture: sterile seeds were sown in agar-filled 1.5 ml microcentrifuge tubes without cap and bottom. Microcentrifuge tubes were filled with 1.5 ml agar (0.68%) and tube bottoms cut off after agar had solidified, standing in a rack holder, and placed into sterile Combiness boxes (Microbox, Belgium) contained 300 ml half-strength MS medium. Adding 1 Arabidopsis seed into each tube, the boxes were incubated in a cold room for three days and then transferred to a growth cabinet (21°C, 16 h/8 h day/night, 150 µmol m^−2^ s^−1^). Plant roots grew from tubes into the solution. The boxes were aerated from day 11 after sowing by pumping air through a sterile filter (0.22 µm Millipore Filter, Ireland). Plants were grown for another 20 days and then in N-free MS medium for 3 days. Then 20 ml of ^GFP^ *E. coli* (OD_600 nm_ = 30) was added for 24 h. Plant incubated with 20 ml of wash buffer were used as a control. Plant were harvested, rinsed in deionized water, and immediately submersed in liquid N_2_ and stored at −80°C.

Tomato (*Solanum lycopersicum*) vermiculite culture: seeds were geminated in soil for 10 days prior to being transferred into 200 ml pots containing vermiculite (one seedling per pot) in a growth room (16/8 h light/dark, 21°C, 150 µmol m^−2^ s^−1^). Pots were watered daily with tap water with addition of fertilizer (N-P-K: 15-15-15) once a week. Plants were grown for 2 to 3 weeks until shoot size was 10–15 cm.

Tomato hydroponic culture: 8–12 cm tall plants grown on vermiculite were carefully transferred into hydroponic culture consisting of 0.5 l water at pH 5.8 supplemented with 10 µM CaSO_4_ (hydroponic solution) with 1 seedling per pot. The hydroponic cultures were continuously aerated and mixed by gentle stirring with a magnetic stirrer bar.

### Uptake of *E. coli* and Yeast by Roots of Arabidopsis and Tomato

To assess uptake of *E. coli* and yeast by Arabidopsis, 5 ml of ^GFP^ *E. coli* or ^GFP^yeast preparation (see above) at a cell density of 2 *A*_600_ units was carefully added to roots of plants grown axenically on MS plates (see above) and incubated for 4 h horizontally at room temperature. Plants were carefully removed from the medium and roots washed with deionized water before being analyzed by confocal laser microscopy (CLSM, see details below). To assess uptake of *E. coli* and yeast by tomato in hydroponic cultures, plants were initially grown in hydroponic solution for 3 days to ensure the integrity of the roots. 20 ml of ^GFP^ *E. coli* or ^GFP^yeast preparation at a cell density of 50 *A*_600_ units was then added into the 500 ml hydroponic culture. After an overnight incubation at room temperature, roots were washed with deionized water and analyzed by CLSM.

For analysis of Arabidopsis and tomato root section by CLSM, visually assessed roots regions showing high fluorescence were excised (5–10 mm long), washed and embedded in 3% agarose. Hand-cut cross sections were transferred into curved slides, washed thoroughly with deionized water and analyzed by CLSM (see below). For analysis of tomato root sections by TEM, root regions showing high uptake by CLSM were coated with agarose before processing to ensure that bacteria external to roots were trapped in the agarose and not dislodged during cutting.

### Time Course Experiment to Assess Status of ^GFP^yeast in Tomato Roots

Ten tomato plants grown hydroponically for three days in hydroponic solution (see above) were incubated with ^GFP^yeast overnight. Roots were carefully rinsed with deionized water and plants were placed in fresh hydroponic solution. The hydroponic solution was replaced every two days. Duplicate plants were removed from hydroponic culture at different time points and roots were treated with hydrogen peroxide (15%, 10 min) to sterilize the root surface. Roots from one plant were analyzed by CLSM and roots from the other were ground in liquid N_2_ and analyzed for TDH3:GFP content by western blotting.

### Western Blotting

Entire tomato roots were ground in liquid nitrogen and resuspended in 0.5 ml of 50 mM Tris (pH 7.5) supplemented with 0.1% Tween20. Non-soluble material was discarded by centrifugation at 14,000 rpm for 30 min. Total protein content of the extracts was determined as described by Bradford [48]. Equal amounts of protein sample was resolved by SDS-PAGE and characterized by western blot analysis using anti-GFP antibody (0.4 µL ml^−1^, Roche) as primary antibody and Alexa Fluor 680 goat anti-mouse (Molecular Probes) as secondary antibody. Detection was performed with an Odyssey infrared imaging system (Li-COR, USA).

### ^15^N-Labeling of *E. coli*

^15^N-labeling of *E. coli* Bl21 cells was carried out as described by [49]. ^15^N-labeled *E. coli* cells (0.5 l) were harvested by centrifugation and washed four times with 0.5 l of deionized water. *E. coli* cells were then re-suspended in 1 l of water and used immediately for the incubation experiment.

### Uptake of ^15^N-Labeled *E. coli* by Tomato

Twenty-one tomato plants (15 days old) were grown for three days in hydroponic solution (see above). Seven plants were incubated in 1 l of ^15^N-labeled *E. coli* solution for 1 h. After incubation, roots were gently rinsed with deionized water and plants were transferred to hydroponic solution. During this process, special care was given to avoid any contamination of the shoots by the bacterial solution. The remaining ^15^N-labeled *E. coli* incubation solution was centrifuged (3000 rpm, 15 min) and the supernatant sterilized by filtration (0.22 µm Millipore Filter, Ireland) to remove remaining *E. coli* cells. Seven plants were incubated for 2 h in the filtered supernatant (“control 2”). After incubation, roots were gently rinsed with sterile deionized water and plants were further grown in hydroponic solution. A further seven plants were grown in hydroponic culture without addition of *E. coli* (“control 1”). All plants were grown for a further 2 weeks with hydroponic solution changed daily. Subsequently, 2–3 new leaves of each plant were excised and dried at 60°C overnight, weighted and homogenized. The samples were analyzed for total nitrogen (N) and ^15^N content with continuous flow Isotope Ratio Mass Spectrometer (IRMS, Stable Isotope Facility, University of California, Davis).

### Microarray Analysis

Total RNA of Arabidopsis roots grown in hydroponic culture were extracted using NucleoSpin® Plant Kits (BD Biosciences Clontech, Japan). RNA of plants incubated with or without *E. coli* (control) were labelled with Cy3 or Cy5 fluorescent dye, mixed and used for subsequently hybridization onto 4x44K Agilent Arabidopsis GeneChip arrays (Agilent Technologies, USA). Labelling and hybridization of RNA, including scanning of the chips were performed by the Australian Genome Research Facility (AGRF, Victoria, Austrlia). Expression values (log10) for three biological replicates were extracted using robust multi-array analysis with perfect match correction and quantile normalization. Genes with ≥3 fold change were computed using one-way ANOVA (p<0.05) with Partek Genomics suite.

### Accession Numbers

The microarray hybridization data have been submitted to the National Center for Biotechnology Information (NCBI) Gene Expression Omnibus (http://www.ncbi.nlm.nih.gov/geo) under accession number GSE22277.

### Electron Microscopy

Roots of Arabidopsis and tomato incubated with *^GFP^E. coli* or *^GFP^*yeast were fixed in 4% paraformaldehyde in 0.1 M phosphate buffer pH 6.8 overnight at 4°C. After washing in 0.1 M phosphate buffer, roots were dehydrated through a graded ethanol series and infiltrated with LR White Resin and polymerized overnight at 50°C. Thin sections were cut with a Leica Ultracut UC6 ultramicrotome, picked up on carbon coated copper grids, stained with uranyl acetate and Reynold's lead citrate [50] and viewed in a JEOL 1010 transmission electron microscope operated at 80 kV and images were captured on a Olympus Soft Imaging Solutions Megaview III digital camera.

### Gold Labeling

Thin sections were labeled using an anti-GFP antibody (Clontech, Mountain View, USA) as the primary antibody and a goat anti-mouse secondary labeled with 10 nm colloidal gold (British Biocell International, Cardiff, UK). Sections were also labeled with cellulase gold, made according to [51]. The cellulase was 1,4-(1,3:1,4)-β-D-Glucan 4-glucano-hydrolase from *Trichoderma reesei* (Sigma Aldrich, St Loius, USA). As a control, root sections were exposed to 2 mg ml^−1^ cellulase for 16 h prior to labeling.

### Cellulase Activity Analysis

26-days old hydroponically grown Arabidopsis were incubated with *E. coli* Bl21 overnight. Plants not incubated with *E. coli* were used as negative control. Roots were rinsed twice in fresh medium and then transferred to fresh medium containing 31 µg ml^−1^ resorufin-β-D-cellobioside (Res-CB) (Marker Gene Technologies Inc., Eugene, OR, USA), a long-wavelength fluorescent substrate, which releases red fluorescent fluorophore resorufin upon cleavage. Roots were incubated for 2 h at room temperature, washed and inspected under CLSM.

### Confocal Microscopy

A Zeiss LSM510 META (Carl Zeiss, Germany) confocal laser scanning microscope (CLSM) was used with 10x dry, 20x water immersion objectives, 40x and 60x oil immersion objectives. GFP and Res-CB were visualized by excitation with an argon laser at 488 nm and HeNe1 laser at 543 nm; detection with a 505–530 nm and 560–615 nm band-path filter, respectively.

## Supporting Information

Figure S1 Roots of Arabidopsis (A) and tomato (B) plant incubated with nano-silica fluorescent beads. No nano-beads were detected inside roots. Bar corresponds to 50 µM.
(5.72 MB TIF)

Figure S2 Roots of tomato plant incubated with yeast expressing GFP.
(5.27 MB TIF)

Figure S3 Arabidopsis grown with or without *E. coli* Bl21 incubation maintained a healthy phenotype.
(7.36 MB TIF)

Figure S4 Arabidopsis genes involved in the cytoskeleton structure and re-organization with differential expression at the time incubated with *E. coli* Bl21 compared with control.
(9.60 MB TIF)

Movie S1 Presence of *E. coli* inside root hairs of Arabidopsis incubated with *E coli* containing green fluorescent protein.
(7.99 MB AVI)

Movie S2 Presence of yeast inside root hairs of Arabidopsis incubated with yeast containing green fluorescent protein.
(7.99 MB AVI)

Movie S3 Presence of *E. coli* inside roots of tomato incubated with *E coli* containing green fluorescent protein.
(5.59 MB AVI)

Movie S4 Tomato root transverse sections show ^GFP^ *E. coli* in the apoplast and inside the root cells.
(9.59 MB AVI)

Movie S5 Tomato root transverse sections show ^GFP^yeast in the apoplast and inside the root cells.
(6.43 MB AVI)
