# Supplementary figures and images for: Turning the Table: Plants Consume Microbes as a Source of Nutrients
Source: PLoS One. 2010 Jul 30;5(7):e11915. doi: 10.1371/journal.pone.0011915 (PMC2912860; doi:10.1371/journal.pone.0011915)

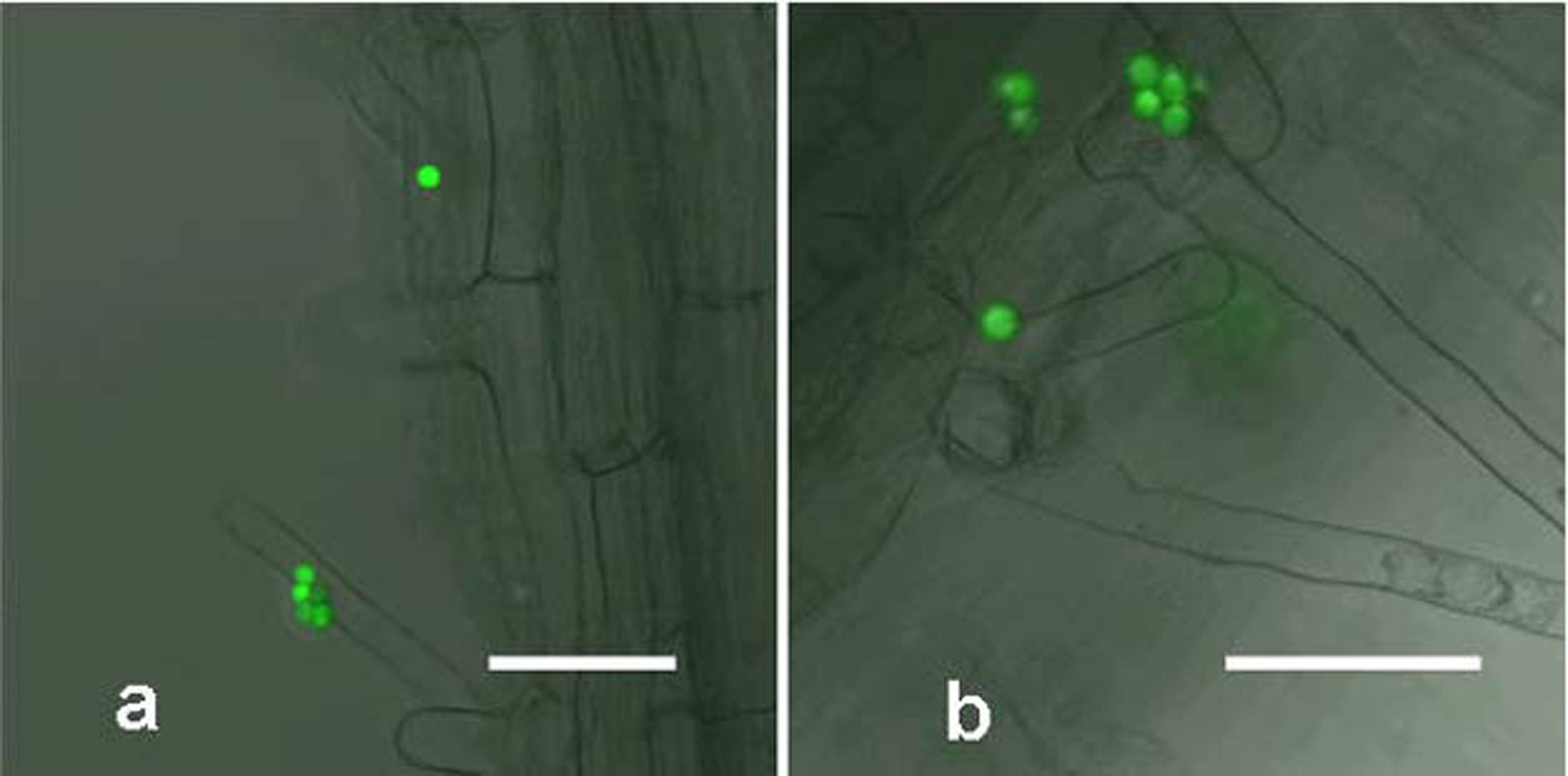

Supplement: Figure S1 — Roots of Arabidopsis (A) and tomato (B) plant incubated with nano-silica fluorescent beads. No nano-beads were detected inside roots. Bar corresponds to 50 µM. (5.72 MB TIF) [file pone.0011915.s001.tif]

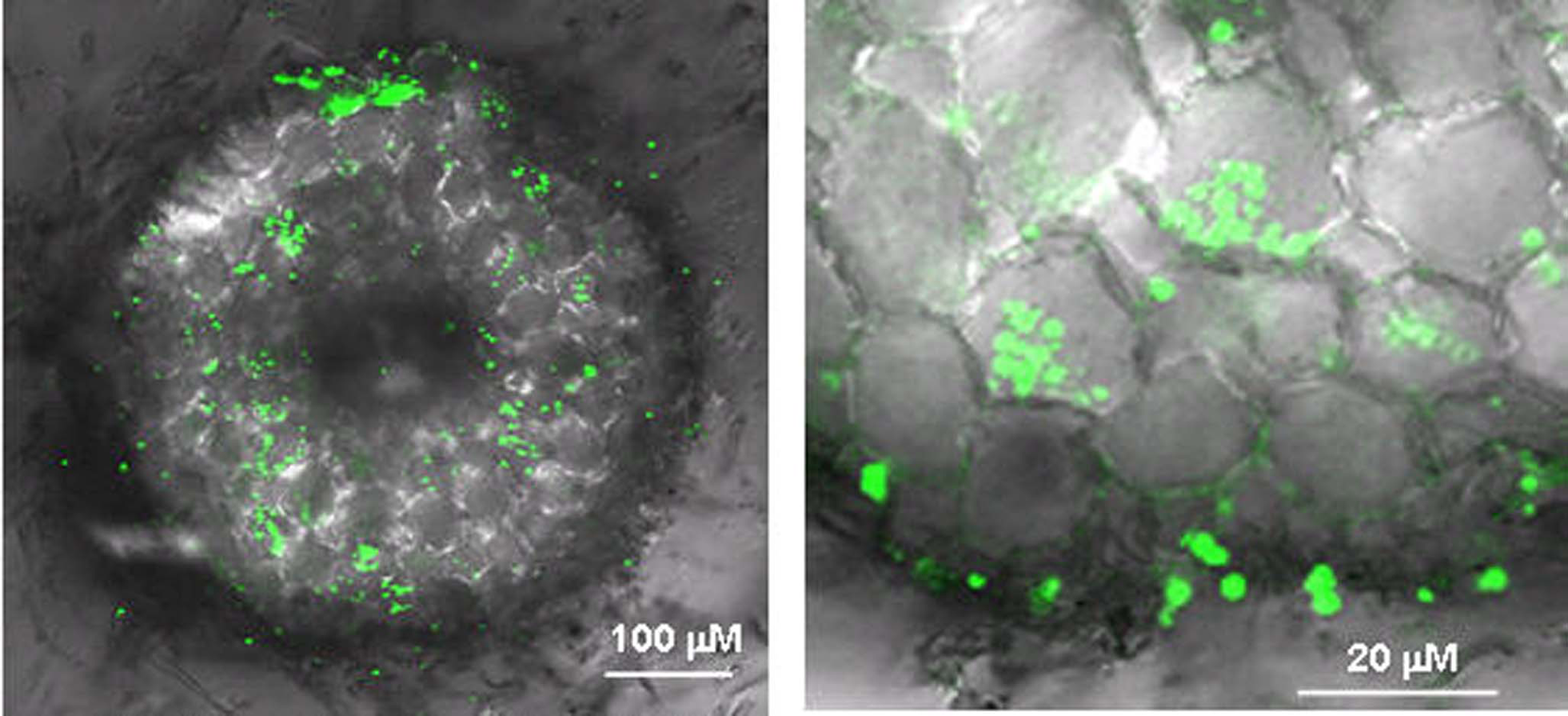

Supplement: Figure S2 — Roots of tomato plant incubated with yeast expressing GFP. (5.27 MB TIF) [file pone.0011915.s002.tif]

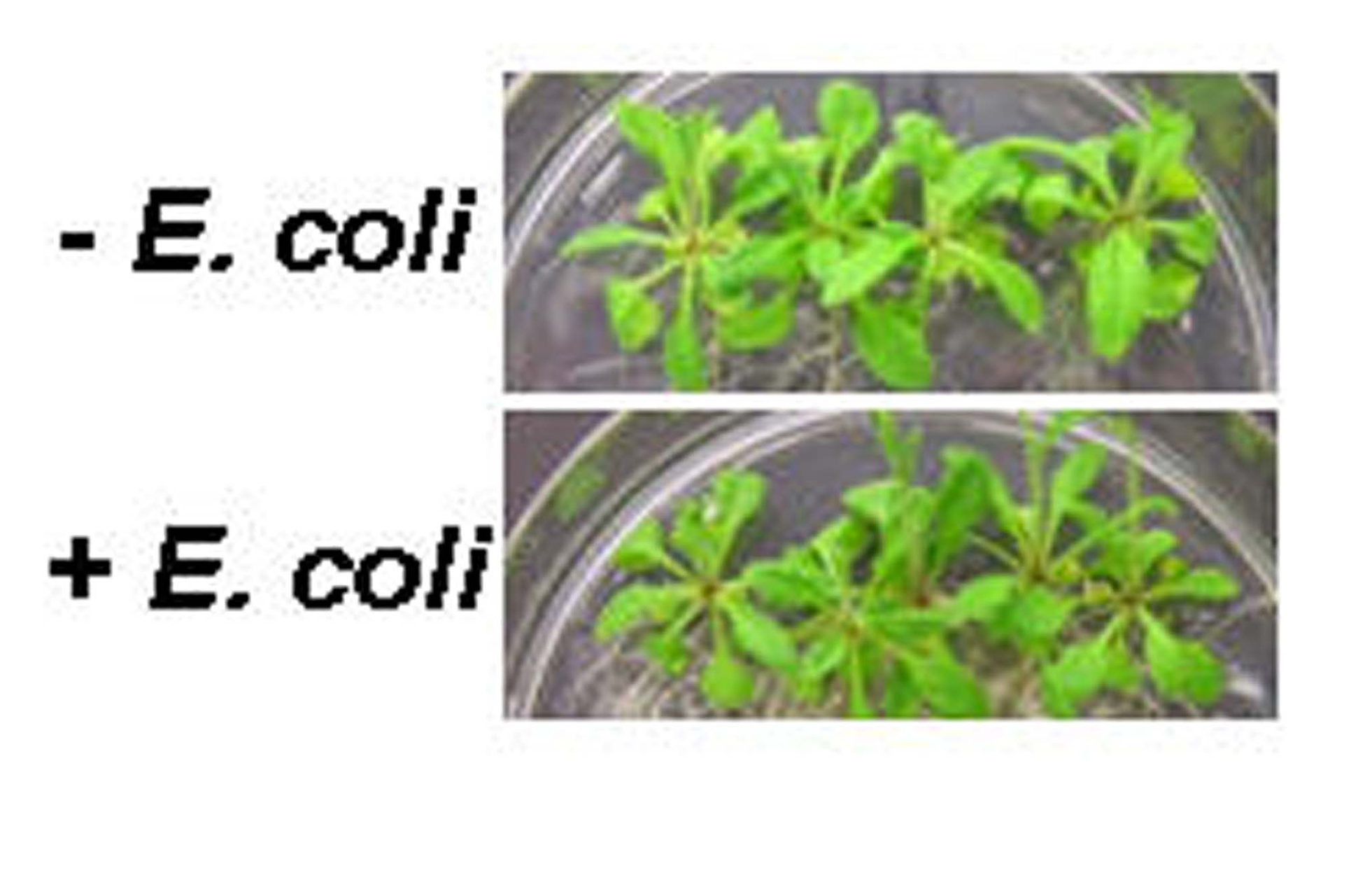

Supplement: Figure S3 — Arabidopsis grown with or without E. coli Bl21 incubation maintained a healthy phenotype. (7.36 MB TIF) [file pone.0011915.s003.tif]

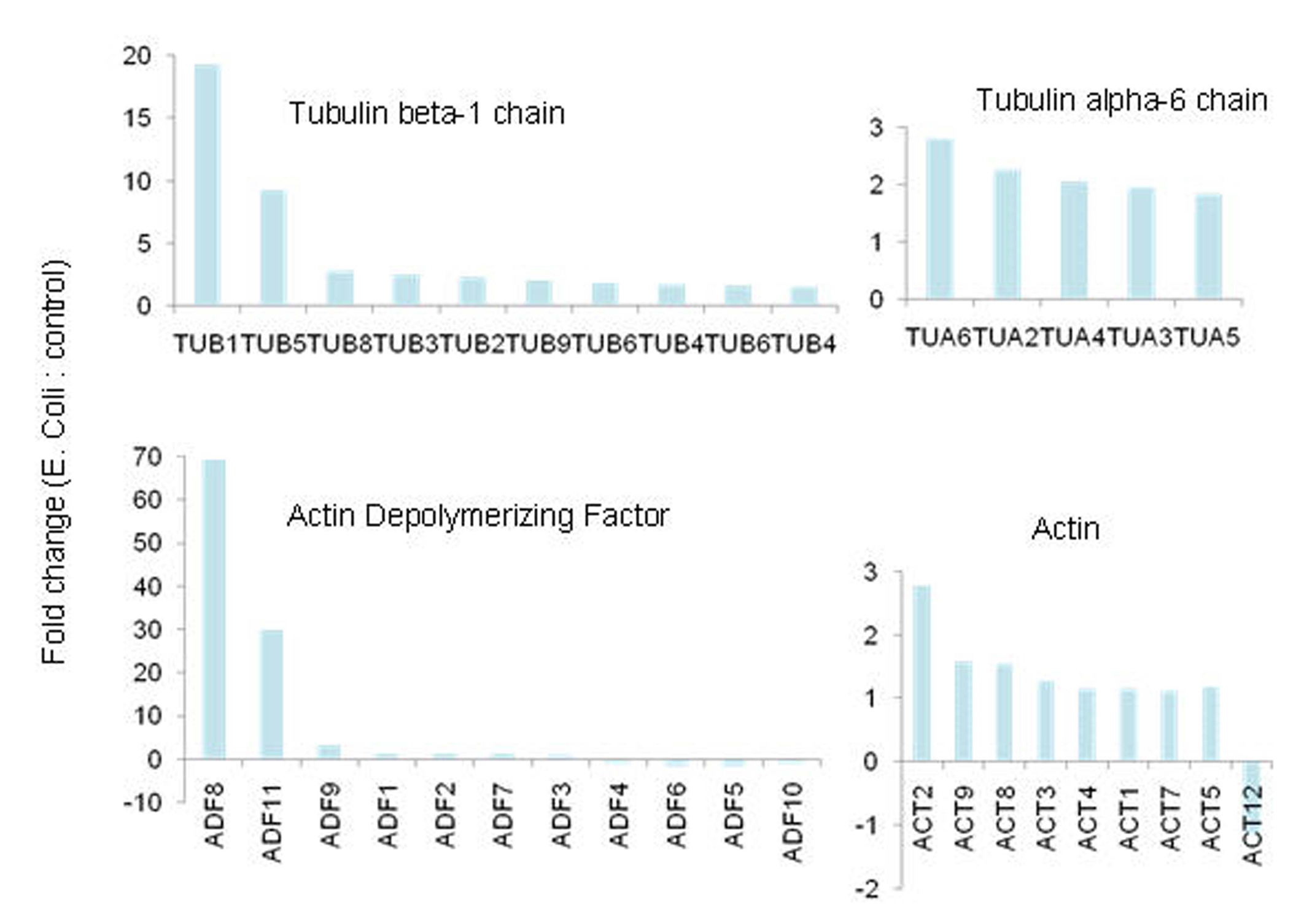

Supplement: Figure S4 — Arabidopsis genes involved in the cytoskeleton structure and re-organization with differential expression at the time incubated with E. coli Bl21 compared with control. (9.60 MB TIF) [file pone.0011915.s004.tif]
